# Prognostic Factors for Survival and Relapse in ANCA-Associated Vasculitis with Renal Involvement: A Clinical Long-Term Follow-Up Study

**DOI:** 10.1155/2018/6369814

**Published:** 2018-10-16

**Authors:** Anna Salmela, Tom Törnroth, Tuija Poussa, Agneta Ekstrand

**Affiliations:** ^1^Department of Internal Medicine, Vaasa Central Hospital, Vaasa, Finland; ^2^Department of Pathology, Helsinki University Hospital, Helsinki, Finland; ^3^STAT-Consulting, Nokia, Finland; ^4^Abdomen Center, Nephrology, Helsinki University Hospital, Helsinki, Finland

## Abstract

**Aim:**

We describe the clinical pattern of ANCA-associated vasculitis (AAV) and assess long-term prognostic factors of patients and renal survival and relapse.

**Methods:**

Data from 85 patients with renal biopsy-proven AAV at a single center with up to 20-year [median 16.2 years (95% CI 14.9-17.7)] follow-up were retrospectively collected.

**Results:**

Overall, 55% of the patients had microscopic polyangiitis (MPA) and 45% had granulomatosis with polyangiitis (GPA). The histopathological classes were focal in 35%, crescentic in 26%, mixed in 20%, and sclerotic glomerulonephritis in 19% of the patients. As induction treatment, a combination of cyclophosphamide and corticosteroids was given to 82%, while a combination of azathioprine and corticosteroids was maintenance therapy in 79%. The twenty-year patient survival was 45%. In a multivariable analysis, age ≥58 years [hazard ratio (HR) 7.64, 95% CI 3.44-16.95] and myeloperoxidase (MPO) ANCA (HR 2.12, 95% CI 1.08-4.17) were associated with shorter patient survival time. Renal survival was 68% overall: 88% in focal, 71% in crescentic, 56% in mixed, and 37% in sclerotic class (p=0.01). Female sex (HR 0.26, 95% CI 0.10-0.73) was a predictor of improved renal survival, whereas GFR <30 ml/min and MPO-ANCA were associated with worse renal survival (HR 4.10, 95% CI 1.35-12.49 and HR 3.10, 95% CI 1.21-7.95, respectively). Relapse-free survival at 20 years was 10%. MPA was associated with a lower risk for relapse (HR 0.48, 95% CI 0.28–0.82).

**Conclusion:**

We confirmed the improved patient and renal survival in AAV patients with glomerulonephritis, while relapse remained the primary challenge. Histopathological classification may be relevant for survival.

## 1. Introduction

Granulomatosis with polyangiitis (GPA) and microscopic polyangiitis (MPA) are the primary types of vasculitis that are associated with anti-neutrophil cytoplasm antibody (ANCA). Renal vasculitis is the most common severe manifestation of ANCA-associated vasculitis (AAV) typically presented with rapidly progressive glomerulonephritis (GN). Dialysis is often needed during the AAV diagnostic phase, although renal recovery and withdrawal from dialysis after treatment may occur. However, treatment per se may cause significant morbidity, and patients with impaired renal function may be particularly prone to treatment-emergent adverse events [1]. Renal impairment at diagnosis also predicts poor renal [2–5] and patient survival [6, 7].

Medication based on cyclophosphamide (CYC) and corticosteroids (CS), which have been used since the 1970s [8], changed AAV prognosis from lethal to a chronic relapsing disease. Approximately one-half of the patients experience a relapse within five years after diagnosis [9, 10].

Biopsy-proven pauci-immune necrotizing GN is a gold standard for the diagnosis of renal AAV. In 2010, a histopathologic classification with focal, crescentic, mixed, and sclerotic categories of GN in AAV (AAGN) was introduced [2].

The aim of the present study was to describe the clinical profile at diagnosis and the long-term outcome of newly diagnosed, biopsy-proven renal AAV patients during a follow-up of 20 years at a tertiary clinic. We also assessed prognostic factors, including histological classification, which influence patient and renal survival and relapse.

## 2. Material and Methods

### 2.1. Study Design

This longitudinal, retrospective cohort study was performed at the Division of Nephrology at the Helsinki University Hospital. The catchment area was Helsinki and Uusimaa Hospital District with 1.5 million inhabitants. The study was approved by the Department of Internal Medicine of the hospital. Since the data were retrospectively gathered from medical records, informed consent from the patients was not required, and permission from the Ethics Committee was not needed.

### 2.2. Patients and Clinical Data

All consecutive patients diagnosed with kidney biopsy-proven ANCA-positive vasculitis between 1996 and 2005 were included. The patients were divided into MPA and GPA clinical diagnostic groups according to the European Medicines Agency algorithm [11]. Renal-limited vasculitis (RLV) was considered as a form of MPA. Finally, the 2012 Chapel Hill Consensus Conference nomenclature was applied [12].

Patients' medical records were systematically reviewed from diagnosis until December 31, 2017, death, or loss to follow-up. The time of diagnosis was defined as the date of admission to the Department of Nephrology. The duration of AAV symptoms before diagnosis was recorded as self-reported by the patient. For ANCA-testing, ANCA specificity against myeloperoxidase (MPO-ANCA) and proteinase 3 (PR3-ANCA) obtained from the enzyme-linked immunosorbent assays (ELISA) were used. A kidney biopsy was performed in all patients within 1 week after admission.

Kidney function was measured using the estimated glomerular filtration rate (GFR) determined by the CKD-EPI formula [13]. GFR was recorded at the time of diagnosis and at 1, 3, 5, 10, 15, and 20 years. End-stage renal disease (ESRD) was defined as the need for permanent dialysis or transplantation. Patient survival, renal survival, and relapses were recorded until the end of follow-up.

Disease activity and organ involvement in vasculitis were measured using the Birmingham Vasculitis Activity Score (BVAS) [14]. Remission was defined as the absence of disease activity (BVAS 0). Relapse was defined as the worsening of AAV clinical disease activity requiring augmentation of immunosuppressive treatment.

### 2.3. Histopathological Classification of Renal Biopsy

Renal histology was re-evaluated by a single pathologist (TT) and categorized into four classes of AAGN: (A) focal (at least 50% of glomeruli were normal), (B) sclerotic (at least 50% of glomeruli were sclerotic), (C) crescentic (at least 50% of glomeruli presented with cellular crescents), and (D) mixed (not suitable with previous criteria) [2].

### 2.4. Treatment Schedule

Induction treatment was administered according to the local treatment protocol based on a combination of CYC and CS. CYC was administered either orally (from 2 mg/kg/d) or intravenously (0.75 g/m^2^ every 2–3 weeks). CYC was administered for 3–6 months until stable remission was achieved. The initial CS dose was 1 mg/kg/day followed by dose tapering, aiming for 10 mg/day at 6 months. Intravenous pulses of 500–1000 mg methylprednisolone for 3 days were administered in cases exhibiting rapidly deteriorating renal function or other severe manifestation. Plasma exchange was considered for patients with alveolar hemorrhage or severe renal failure (GFR <15 ml/min). The treatment considered individually according to the clinical situation of the patient, taking into account the severity of the disease and the possible risks of the treatment for the patient.

Maintenance treatment was based on azathioprine (AZA) combined with low-dose CS.

### 2.5. Statistics

The associations between diagnoses and histopathological groups vs. clinical baseline characteristics were assessed using the Kruskal–Wallis and Mann–Whitney* U* tests for continuous data and the Chi-squared and Fisher's exact tests for categorical data. Bonferroni corrections were used when appropriate. The Spearman rank correlation (Rho) was used to study the association between continuous variables. The three primary endpoints were as follows: (i) patient survival, defined as the time from the diagnosis to death; (ii) renal survival, defined as the time from the diagnosis to ESRD; and (iii) relapse-free survival, defined as the time from the diagnosis to the first relapse. The Kaplan–Meyer method was used to draw survival curves and estimate the survival rates at 1, 5, 10, 15, and 20 years of follow-up and median survival times with 95% confidence intervals. Patients without documented events were censored at last follow-up time. The median follow-up time was assessed using the reverse Kaplan-Meier method.

Potential prognostic factors were diagnosis, ANCA specificity, histopathological class, sex, age, proteinuria, GFR, and CYC treatment. Univariate Cox proportional hazard models were used to assess the associations between these potential prognostic factors and the endpoints. Multivariate analyses were then performed to find the most important predictors. The models used the forward-stepping covariate selection procedure. Variables that were significant or almost significant (p<0.10) predictors in univariate models were introduced into the models. At each step, the criterion for entry was p<0.10. The results are expressed as the HR with 95% CI. Friedman's two-way ANOVA was used to analyze GFR repeated measurements, and Bonferroni corrections were used to analyze the differences between time points. The Mann–Whitney U test was used to test the difference between diagnoses of GPA vs. MPA, and the Kruskal–Wallis test with Bonferroni pairwise corrections was used to test the differences between histopathological subgroups. If the patient was on dialysis, the GFR was estimated using a value of 2.5 (i.e., 5.0/2, where 5.0 was the minimum GFR in nondialysis patients). All statistical tests were two-sided, and P-values <0.05 were considered to be statistically significant. Analyses were performed using IBM SPSS Statistics for Windows (version 25.0, Armonk, NY, USA, IBM Corp.), and figures were created using SigmaPlot version 13.0 (Systat Software, Inc., San Jose California USA).

## 3. Results

**Figure 1:**
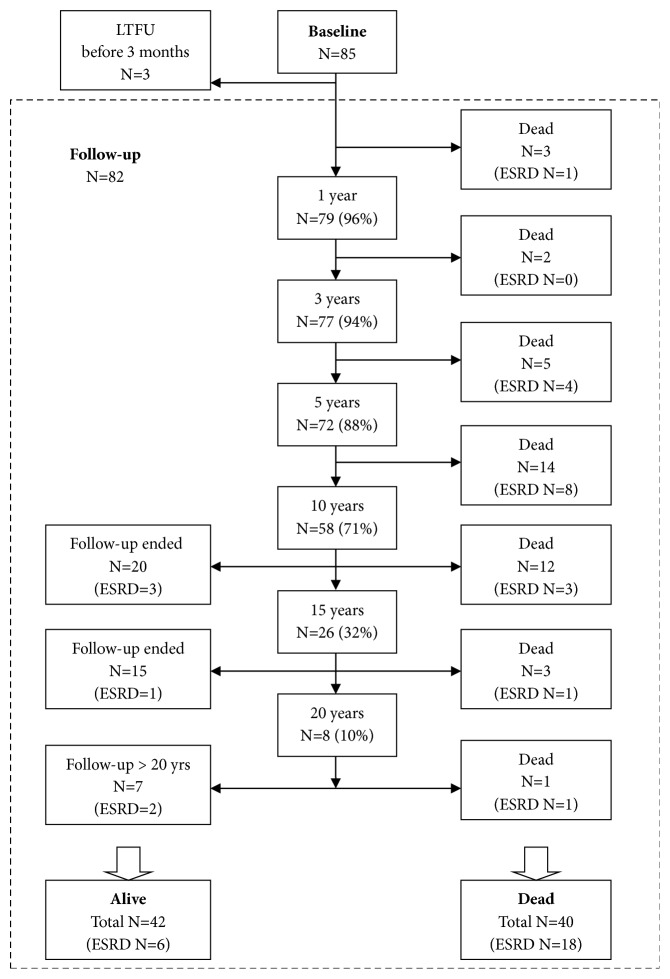
Patient flowchart.

Overall, 85 patients were diagnosed with ANCA-positive vasculitis with renal biopsy verification. Time from the beginning of the symptoms to diagnosis was 4 months (IQR 2–6), with no significant difference between MPA (median 4, IQR 3–6) and GPA (4, 2–8; p=0.61). Three patients were lost to follow-up within 3 months of diagnosis, and therefore, they were excluded from the follow-up data analysis. The patient flow of the study is presented in Figure 1.

### 3.1. Baseline

Demographics of the 85 patients at baseline are presented in Table 1. The median age of patients at baseline was 58 years (range 22-80) and 10.6% (9/85) were over 75 years of age.

Within 1 month after diagnosis, 13 patients (15.3%) were on dialysis with no difference between MPA and GPA (17.0% vs. 13.2%, p=0.62). Renal function did not correlate with the duration of symptoms before diagnosis (Rho 0.16, p=0.14).

A renal histology report was available for 84 patients. The median number of glomeruli in the biopsy was 15 (range, 8–50). The patient distribution across the AAGN classes was as follows: 34.5% (29/84) were focal, 26.2% (22/84) were crescentic, 19.0% (16/84) were sclerotic, and 20.2% (17/84) were mixed. The histopathological classes according to diagnosis and ANCA specificity are shown in Table 2.

### 3.2. Follow-Up

The median follow-up time was 16.2 years (95% CI 14.9-17.7). All patients, except two who died early, attained remission within a median time of 3 months (range 1–12).

### 3.3. Treatment

Most of the patients (81.7%, 67/82) were initially treated with a combination of CYC and CS. CYC was administered intravenously to 61.2% (41/67) and orally to 38.8% (26/67) of the patients. The median cumulative CYC oral dose was 13250 mg (IQR, 7350–20900) and 9500 mg (7000–12000) for the cumulative CYC intravenous dose (p=0.01). In addition, 8.5% of the patients were treated with plasmapheresis, and 3.7% were administered intravenous immunoglobulins. AZA and CS or CS alone were used for remission induction in 11.0% and 4.9% of the patients, respectively.

In general, the patients who were not treated with CYC were older than those treated with CYC (median 71 vs. 53 years, p<0.001), had lower GFR (four patients needed dialysis; GFR 15 vs. 27ml/min, p=0.09) and renal histology was either dominated with sclerotic features or without active vasculitic lesions. Among the MPA patients, 68.9% (31/45) and 97.3% (36/37) of GPA patients were treated with CYC (p=0.001). CYC treatment was associated with histology (p=0.003) as follows: 53.3% of the patients with sclerotic AAGN, 82.4% with mixed AAGN, 85.2% with focal AAGN, and 100% with crescentic AAGN were treated with CYC (sclerotic vs. crescentic AAGN, p=0.002).

Maintenance therapy was administered to 96.3% (77/80) of the patients. The most common combination was AZA and CS in 79.2% (61/77) of the patients. Other maintenance medications used were CS alone in 10.4% or in combination with mycophenolate mofetil (MMF) in 5.2% or CYC in 3.9% of the patients.

### 3.4. Patient Survival and Causes of Death

**Figure 2:**
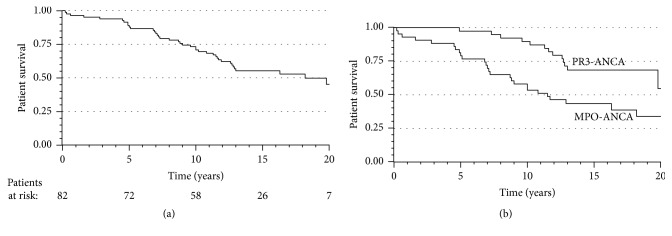
(a) Kaplan-Meier patient survival curve for all patients. (b) Kaplan-Meier patient survival curve for ANCA subgroups. The univariate Cox proportional hazards model: HR (95% CI) 2.67 (1.37-5.19), p=0.004, when the MPO-ANCA-positive patients were compared to PR3-ANCA-positive patients.

The cumulative patient survival after 20 years was 45% (95% CI 31.2-59.2%) as shown in Figure 2(a). Accordingly, the 1-year mortality was 4% (3 deaths), 5-year mortality was 12% (10 deaths), 10-year mortality was 29% (24 deaths), 15-year mortality was 45% (36 deaths) and 20-year mortality was 55% (40 deaths); in addition, the last death occurred at 20.2 years. The cause of death was available from 82.5% (33/40) of the patients; of deaths, 9.1% (3/33) were directly related to vasculitis activity. The main causes of death within the first year were infections (in two patients) and intracerebral bleeding (in one patient). Within years two to five of follow-up, three out of seven deaths were caused by acute myocardial infarction; other causes were infection, intracerebral bleeding and cancer. Later during the follow-up, the main causes of death were infections, malignancies and cardiovascular diseases. Of all deaths, 18 (45%) occurred in patients with ESRD. Five patients died with a functioning kidney transplant; four of these deaths were caused by malignancies and one was infection.

The median survival time was 18.2 years (95% CI 13.2-23.2). The results from the univariate and multivariate Cox regression analysis predicting patient survival are presented in Table 3. The 20-year survival of patients treated with CYC was better than those patients who were not treated with CYC (48% vs. 30%, respectively; HR 0.42, 95% CI 0.20-0.88; p=0.02). However, in a multivariate analysis, age ≥58 years and MPO-ANCA were the only significant predictors of shorter survival (Figure 2(b)).

### 3.5. Kidney Function and Renal Survival

**Figure 3:**
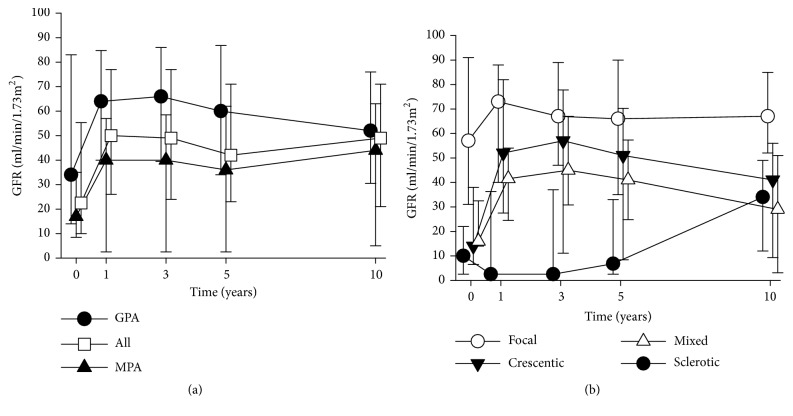
(a) Median GFR during follow-up in diagnostic groups GPA and MPA. For up to 5 years, the difference between the diagnostic groups was significant (p<0.02, Mann–Whitney U test). (b) Median GFR during follow-up in renal histology categories. For up to 5 years, the difference between histology categories was significant (p≤0.001, Kruskal–Wallis test), and the Bonferroni-corrected pairwise comparisons detected a significant difference between the focal and sclerotic AAGN (p<0.001). The error bars indicate the interquartile range.

Changes of GFR during the follow-up are shown in Figure 3(a). Due to deaths and the end of the follow-up cases after 10 years, the number of GFR measurements decreased and therefore were not included in the analysis. GFR was higher in GPA than in MPA at all measurement points between diagnosis and 5 years (p<0.02 at 0-5 years, Figure 3(a)). During follow-up visits, until 10 years, GFR was significantly related to histopathological class (p≤0.001). Bonferroni-corrected pairwise comparisons indicated that kidney function in patients with focal AAGN remained the best compared with that in the sclerotic class, where kidney function remained low until the 5-year follow-up visit (p<0.001) (Figure 3(b)). Of note, after 1 year, the higher mortality of those with low GFR causes an overestimation of GFR during the follow-up time, which explains the unexpected appearance of a rising GFR.

**Figure 4:**
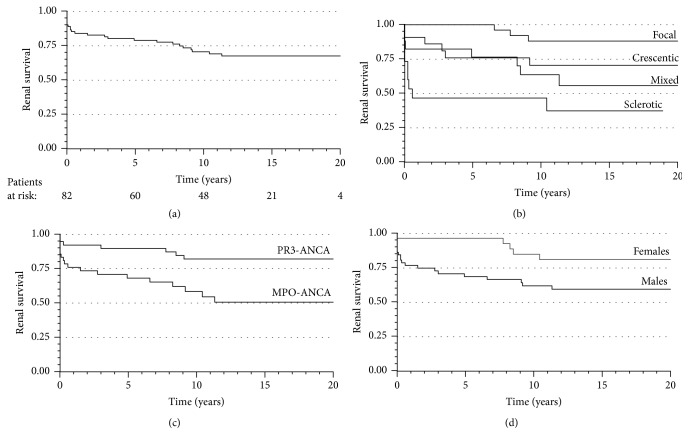
(a) Kaplan-Meier renal survival curve for all patients. (b) Kaplan-Meier renal survival curve for histology groups. The univariate Cox proportional hazards model HR (95% CI) 0.11 (0.03-0.41), p=0.001 in focal; 0.32 (0.11–0.90), p=0.03 in crescentic; and 0.49 (0.18–1.33), p=0.16 in mixed AAGN when sclerotic AAGN was included as a reference. (c) Kaplan-Meier for renal survival for ANCA subgroups. The univariate HR (95% CI) was 3.17 (1.31-7.64), p=0.01, when the MPO-ANCA-positive patients were compared to PR3-ANCA-positive patients. (d) Kaplan-Meier renal survival curves for female and male. The univariate Cox proportional hazards model: HR (95% CI) 0.36 (0.14-0.96), p=0.04, when the female patients were compared to male patients.

Among the 13 patients who were on dialysis within 1 month after diagnosis, five had reversible renal failure and were withdrawn from dialysis. Overall, 25 patients developed ESRD. One-year renal survival was 84% (95% CI 76.2-97.0), 5-year was 79% (95% CI 70.1-87.9) and 10-year was 71% (95% CI 60.5-80.8). After 10 years, two cases of ESRD occurred, with the last one at 11.3 years. The overall renal survival was 68% (95% CI 57.1-78.2) (Figure 4(a)). Among the patients with ESRD, 7 were alive upon renal replacement therapy (RRT) (3 on dialysis, 4 with a kidney transplant), and 18 died (72%) while on RRT (13 on dialysis and 5 with a functioning kidney transplant).

The renal survival rate according to histopathological class was as follows: 88.1% in focal, 70.6% in crescentic, 55.8% in mixed and 37.3% in sclerotic AAGN (p=0.01, Figure 4(b)). According to the post hoc analysis, renal survival was extremely poor in patients with sclerotic AAGN and GFR <15ml/min (N=9) compared with patients with sclerotic AAGN and GFR ≥15ml/min (N=6) (11.1% vs. 83.3%, respectively, HR 10.71, 95% CI 1.28-89.84; p=0.03). The renal survival of the patients with sclerotic AAGN and GFR ≥15ml/min was not significantly different than patients with focal, crescentic and mixed AAGN pooled together (83.3% vs. 75.8%, respectively, HR 0.70, 95% CI 0.09-5.28; p=0.73).

The results from univariate and multivariate Cox regression analyses predicting renal survival are presented in Table 3. The histopathological classification was predictive of survival by univariate but not by multivariate analysis. The treatment with CYC was not significantly associated with renal survival (71% renal survival in CYC treated vs. 53% in patients not treated with CYC; HR 0.43, 95% CI 0.18-1.04; p=0.06). In a multivariate analysis, GFR<30 ml/min and MPO-ANCA were significant predictors of worse renal survival, whereas female sex predicted better renal survival (Figures 4(c) and 4(d)).

To elucidate the possible reasons for the better renal survival in women, we made a post hoc analysis regarding the gender-specific differences and the following variables were included: age, ANCA specificity, AAGN class, diagnostic delay, baseline GFR, proteinuria (≤3g/day vs. >3g/day) and treatment with CYC (or no CYC). However, no significant differences between men and women were found.

As the incidence of ESRD was highest within the first year after diagnosis, we also analyzed the potential risk factors for progression to ESRD more than 1-year after diagnosis. For that purpose, the predictive effects of the following variables on renal survival were analyzed: gender, age, ANCA specificity, diagnosis, AAGN class, proteinuria, and treatment with CYC and GFR at 12 months. Only GFR <30ml/min at 12 months was found to be predictive for renal survival (HR 13.72, 95% CI 4.26-44.16; p<0.001).

### 3.6. Relapses

**Figure 5:**
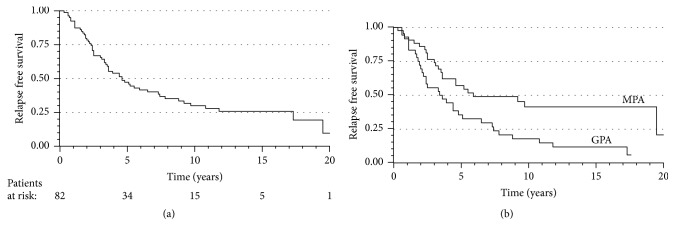
(a) Kaplan-Meier survival curve for relapse-free survival for all patients. (b) Kaplan-Meier survival curves (time without relapse) for GPA and MPA. The univariate Cox proportional hazards model: HR (95% CI) 0.48 (0.28–0.82), p=0.01, when the MPA patients were compared to GPA patients.

Relapse-free survival was 47% (95% CI 36.1-58.4) at 5 years. Thereafter, 10-year relapse-free survival was 30% (95% CI 19.1-40.8), 15-year was 26% (95% CI 15.0-36.6%), and 20-year was as low as 10% (95% CI 0.0-24.7) (Figure 5(a)).

The overall median time to first relapse was 4.6 years (95% CI 3.0-6.2). In patients with GPA, the relapse-free survival was shorter than in patients with MPA (3.4 years, 95% CI 1.3-5.5 vs. 5.9 years, 95% CI 0.9–10.9; HR 0.48, 95% CI 0.28–0.82; p=0.01). Diagnosis was the only significant predictor of relapse-free survival in the multivariate analysis (Table 3; Figure 5(b)).

At the first relapse, 55.4% (31/56) of the patients were without immunosuppression, whereas no relapse occurred in patients on CYC treatment. Of the relapsing patients, 35.7% (20/56) used AZA (with or without GC) at the time of relapse and 8.9% (5/56) were taking only GC. Overall, CYC treatment was not predictive for relapse-free survival (Table 3). No significant differences in relapse rates between the CYC administration routes were found, intravenously vs. orally (HR 1.67, 95% CI 0.90-3.09; p=0.11).

During the 20-year follow-up, 56 patients (68.3%) had at least one relapse. Of all relapsing patients, 57.1% (32/56) had more than one relapse, and the total number of relapses was 129. Of all relapses, 40.3% included renal activity.

AZA combined with GC was used as a relapse treatment in 41.1%, CYC in 23.3%, GC alone in 13.9%, MMF in 12.4%, and methotrexate or cyclosporine in 2.3% of the relapses. From the year 2011 onward, the relapses of five patients (nine relapses altogether) were treated with rituximab (RTX).

## 4. Discussion

This study presents the clinical pattern at diagnosis and the outcome of a 20-year follow-up in patients with renal AAV confirmed with kidney biopsy from a single tertiary center. Twenty-year patient and renal survival were 45% and 68%, respectively. As expected, patient survival was influenced by older age and renal survival by lower GFR. Both patient and renal survival were negatively determined by MPO-ANCA subtype. Furthermore, we found that female sex was related to favorable renal survival. Twenty-year relapse-free survival was 10%, and relapse was more common in patients with GPA.

In our cohort, MPA was more common than GPA. Accordingly, 87% of MPA patients were MPO-ANCA-positive, and 89% of GPA patients were PR3-ANCA-positive. The catchment area of our study was Helsinki and its surroundings in southern Finland, North Europe. Thus, we cannot confirm the observation that GPA or PR3ANCA vasculitis would have a higher incidence in the north [15]. Our results are consistent with the study from southern Sweden, showing a slightly higher incidence of MPA than of GPA [16]. However, MPA dominance in our cohort may be biased because all patients had renal involvement.

The diagnostic delay of 4 months was equivalent in MPA and GPA. This is consistent with recent studies reporting a shortening of the gap between the start of symptoms and diagnosis [6, 16–18]. Despite an equal diagnostic delay, kidney function in MPA was inferior to that in GPA. Accordingly, renal histology showed advanced chronicity in MPA: sclerotic histology was the most prevalent (30%) in MPA patients, whereas in GPA patients, focal (47%) and crescentic (32%) were common. These differences were even more prominent according to ANCA subtype. These results were in accordance with earlier studies showing an association of MPO-ANCA and more chronic histology [3, 4]. In addition, BVAS was higher in GPA than in MPA. These observations may depend on a more indolent course of MPA, which silently results in advanced renal damage.

Since the introduction of the histopathological classification of AAGN in 2010 [2], several groups have validated the prognostic usefulness of this classification in relation to renal outcomes [3–5, 19–25]. Commonly, renal survival has been best in focal AAGN and worst in sclerotic AAGN [3–5, 20, 21, 23, 25]. The median follow-up time of these studies varied between 2.5-8.5 years [3–5, 19–25]. A five-year renal survival time was 66-77% [22, 23, 25], while the 10-year renal survival, reported only by one study, was 60% [22]. In our study, with a median follow-up of 16.2 years, we found renal survival of 79% at 5 years, 71% at 10 years and 68% at 20 years. Renal survival was significantly better in focal (88%) and in crescentic (71%) classes than in sclerotic (37%). It was recently reported that the patients with sclerotic AAGN and GFR lower than 15ml/min had extremely poor renal survival [26] and this result was confirmed in our study. In a multivariate analysis, however, the histopathological group was not predictive of renal outcome. Instead, female sex (HR 0.26) was related to better renal survival, whereas GFR <30 ml/min (HR 4.10) and MPO-ANCA positivity (HR 3.10) were risk factors for ESRD. Although deteriorated GFR is a known predictor of ESRD [2–5, 21–23], there are conflicting results of the effect of MPO (or p-ANCA)-positivity on the renal outcome [3, 5, 16, 21, 23, 25]. Recent studies from Norway also related female gender to favorable renal survival in AAV [27, 28]. The reasons for this gender-specific difference regarding ESRD risk remained elusive in our cohort and further research is required respecting this issue.

Survival of AAV patients has improved continually over the past decades [18, 27, 29]. Five-year survival of renal AAV patients diagnosed after the year 2000 has been 72-84% [29, 30]. In recent studies, 10-year patient survival rates in renal AAV were 49-81% [18, 22]. In our study, 5- and 10-year survival rates were 88% and 71%, respectively. Renal impairment is a known predictor of unfavorable survival [6, 7]. In our study, lower GFR at diagnosis was also related to worse patient survival in the univariate analysis, and 72% of those who reached ESRD died. However, in a multivariate analysis, only ages greater than 58 years (HR 7.64) and MPO-ANCA (HR 2.12) were associated with worse patient survival. Higher age as a negative predictor of survival is expected and consistent with previous results [4, 7, 16]. While the ANCA specificity has mostly not regarded predictive to patient survival [7, 16, 23], a recent, large cluster analysis in AAV patients found a 5.9-fold mortality in renal patients without PR3-ANCA [31].

One explanation for our relatively good long-term patient survival might be the rather low cumulative dose of CYC, which was high enough to induce remission but low enough to minimize complications and mortality. In addition, patient survival is due to good renal survival and short diagnostic delay. The fact that all patients had biopsy-confirmed renal vasculitis comprises a selection bias and may overestimate the patient survival but also confirms that all patients had a severe form of AAV.

Despite improved patient survival over the past decades, the relapse risk has remained high [29]. Consistent with previous studies, we report a relapse-free survival of 47% at 5 years [9, 10]. A recent study of the EUVAS cohort reported a lower 5-year relapse rate of 38% [32]. In that study, a creatinine level > 200 *μ*mol/l was strongly associated with a reduced risk of relapse [32]. In contrast, in our study, the relapse rate remained high and was not influenced by lower GFR; the 20-year relapse-free survival was under 10%, and the only significant predictor of relapse was the diagnosis of GPA. However, the end of a relapse-free survival given by the Kaplan-Meier estimate should be valued with caution due to the large confidence interval. The relapse rate in MPA has been reported to be lower than in GPA [6, 32], but not all have confirmed this result [9, 29]. An upper respiratory tract involvement has been associated with an increased risk of relapse [9]. In our study, we did not examine the effect of an upper airway manifestation on the relapse risk. In GPA patients, ear, nose and throat disease activity were significantly higher, which may contribute to the higher relapse risk compared to that in MPA patients. The relatively low cumulative dose of CYC in our study may also have predisposed the patients to a higher relapse rate compared with other renal cohorts. Somewhat surprisingly, 45% of the patients in our study were on immunosuppressive medication, primarily on AZA, when they relapsed. Rituximab, a monoclonal anti-CD20 antibody, has shown to be superior to AZA in maintaining remission after CYC induction in AAV patients [33]. Rituximab was also given to few patients with multiple relapses in our study.

The strengths of our study are the long follow-up time of 20 years and the diagnosis based on renal biopsy in all patients. The limitations of our study are the retrospective nature and the relatively small number of patients. Also, renal histology was reviewed by a single pathologist.

## 5. Conclusions

In our cohort of renal AAV, a younger age was related to a favorable patient survival, and better GFR at diagnosis was related to improved renal survival, as may be anticipated. MPO-ANCA was a negative predictor of both patient and renal survival. Patients with GPA were more prone to relapse than MPA patients. In addition, we observed that AAGN classification was predictive, as the risk of progressing to ESRD increased with the ascending category of focal, crescentic, mixed and sclerotic AAGN. However, in the multivariate model, the histological class was not associated with renal-free survival. Our cohort confirms the data showing improved patient survival but constantly high relapse risk also in renal patients. The ongoing challenge is to define the factors associated with a higher relapse risk and individualize the maintenance therapy accordingly.

## Figures and Tables

**Table 1:** Baseline characteristics of all patients and diagnostic subgroups.

		MPA	GPA	All	
		N=47	N=38	N=85	P*∗*
Males		27 (57.4)	26 (68.4)	53 (62.4)	0.30
Age (years)		62 (28-80)	52 (22-77)	58 (22-80)	0.004
Hypertonia		14 (29.8)	8 (21.1)	22 (25.9)	0.36
CVD		8 (17.0)	3 (7.9)	11 (12.9)	0.21
Creatinine (*μ*mol/l)		250 (63-2332)	164 (56-1600)	208 (56-2332)	0.02
GFR (ml/min/1.73 m^2^)		17 (1-91)	35 (3-120)	24 (1-120)	0.01
ANCA	PR3-ANCA	6 (12.8)	34 (89.5)	40 (47.1)	<0.001
	MPO-ANCA	41 (87.2)	4 (10.5)	45 (52.9)	
BVAS		15 (11-26)	19 (5-39)	17 (5-39)	<0.001
Proteinuria (g/day)	<0.5	7 (14.9)	6 (15.8)	13 (15.3)	0.31
	0.5-3.0	26 (55.3)	26 (68.4)	52 (61.2)	
	>3.0	14 (29.8)	6 (15.8)	20 (23.5)	
Organ involvement	Renal	47 (100)	38 (100)	85 (100)	
General symptoms*∗∗∗*		33 (70.2)	34 (89.5)	67 (78.8)	0.03
Lung		13 (27.7)	18 (47.4)	31 (36.5)	0.06
ENT		2 (4.3)	23 (60.5)	25 (29.4)	<0.001
Skin		6 (12.8)	9 (23.7)	15 (17.6)	0.19
Eyes/mucous membranes		3 (6.4)	11 (28.9)	14 (16.5)	0.01
Nervous system		3 (6.4)	5 (13.2)	8 (9.4)	0.49*∗∗*
Abdominal		5 (10.6)	1 (2.6)	6 (7.1)	0.22*∗∗*
Cardiac		2 (4.3)	0 (0.0)	2 (2.4)	0.50*∗∗*

Data are presented as the median (range) for continuous nonnormal variables and as the number (%) for categorical variables.

GPA, granulomatosis with polyangiitis; MPA, microscopic polyangiitis (including two patients with renal limited vasculitis); CVD, cardiovascular disease; GFR, glomerular filtration rate; ANCA, anti-neutrophil cytoplasmic antibody; PR3, proteinase 3; MPO, myeloperoxidase; BVAS, Birmingham vasculitis activity score; ENT, ear, nose, and throat.

*∗*P values indicate the comparison between GPA and MPA. Mann-Whitney *U* test was used for nonnormal continuous variables; Chi-squared test and Fisher's exact test (*∗∗*) were used for categorical variables.

*∗∗∗* General symptoms: fever, weight loss, arthralgia, myalgia.

**Table 2:** (A) Histopathological class of AAV in renal biopsies according to diagnosis and ANCA specificity. (B) Baseline characteristics of patients per histopathological class.

			Histopathological class of AAV in renal biopsies					
			Focal	Crescentic	Sclerotic	Mixed	All	
			N=29	N=22	N=16	N=17	N=84	P*∗*
(A)	Diagnosis	MPA*∗∗*	11 (23.9)	10 (21.7)	14 (30.4)	11 (23.9)	46	
		GPA	18 (47.2)	12 (31.6)	2 (5.3)	6 (15.8)	38	0.01
	ANCA	MPO	10 (22.7)	9 (20.5)	14 (31.8)	11 (25.0)	44	0.003
		PR3	19 (47.5)	13 (32.5)	2 (5.0)	6 (15.0)	40	

(B)	Males		17 (58.6)	15 (68.2)	11 (68.8)	9 (52.9)	52 (61.9)	0.71
	GFR, ml/min/1.73 m^2^		60 (5-120)	14 (2-86)	12 (1-38)	21 (5-88)	24 (1-120)	<0.001
	Age, years		55 (23-78)	50 (23-76)	67 (39-80)	60 (22-80)	58 (22-80)	0.02
	Proteinuria >3 g/day		2 (6.9)	4 (18.2)	8 (50.0)	5(29.4)	19 (22.6)	0.01

Data are presented as the median (range) for continuous nonnormal variables and as the number (%) for categorical variables.

AAV, ANCA-associated vasculitis; ANCA, anti-neutrophil cytoplasmic antibody; MPA, microscopic polyangiitis (including two patients with renal limited vasculitis); GPA, granulomatosis with polyangiitis; MPO, myeloperoxidase; PR3, proteinase 3; GFR, glomerular filtration rate.

*∗* Kruskal-Wallis test was used for nonnormal continuous variables and Chi-squared test was used for categorical variables.

*∗∗* The renal biopsy of one MPA patient was not available.

**Table 3:** Diagnostic, histological, and clinical factors to predict patient survival, renal survival, and relapse-free survival (N=82).

			Univariate analysis		Multivariate analysis	
Endpoints	Predictors		HR (95% CI)	P	HR (95% CI)	P
Patient survival	Dg MPA^a^		2.71 (1.37-5.35)	0.004		
	ANCA MPO^b^		2.67 (1.37-5.19)	0.004	2.12 (1.08-4.17)	0.03
	Histopathological class^c^	Focal	0.29 (0.12-0.69)	0.005		
		Crescentic	0.26 (0.10-0.67)	0.006		
		Mixed	0.69 (0.30-1.59)	0.38		
	Female^d^		0.67 (0.34-1.32)	0.25		
	Age ≥58 years^e^		8.50 (3.84-18.79)	<0.001	7.64 (3.44-16.95)	<0.001
	Proteinuria >3g/d^f^		0.94 (0.43-2.04)	0.87		
	GFR <30ml/min/1.73m^g^		2.08 (1.07-4.04)	0.03		
	Treatment with CYC^h^		0.42 (0.20-0.88)	0.02		

Renal survival	Dg MPA^a^		2.71 (1.13-6.52)	0.03		
	ANCA MPO^b^		3.17 (1.31-7.64)	0.01	3.10 (1.21-7.95)	0.02
	Histopathological class^c^	Focal	0.11 (0.03-0.41)	0.001		
		Crescentic	0.32 (0.11-0.90)	0.03		
		Mixed	0.49 (0.18-1.33)	0.16		
	Female^d^		0.36 (0.14-0.96)	0.04	0.26 (0.10-0.73)	0.01
	Age ≥58 years^e^		2.50 (1.10-5.71)	0.03		
	Proteinuria >3g/day^f^		2.46 (1.08-5.60)	0.03		
	GFR <30ml/min/1.73m^2g^		5.63 (1.92-16.49)	0.002	4.10 (1.35-12.49)	0.01
	Treatment with CYC^h^		0.43 (0.18-1.04)	0.06		

Relapse free	Dg MPA^a^		0.48 (0.28-0.82)	0.01	0.48 (0.28-0.82)	0.01
survival	ANCA MPO^b^		0.59 (0.34-1.01)	0.05		
	Histopathological class^c^	Focal	1.29 (0.54-3.08)	0.56		
		Crescentic	1.82 (0.75-4.41)	0.19		
		Mixed	1.07 (0.40-2.82)	0.90		
	Female^d^		1.19 (0.70-2.02)	0.53		
	Age ≥58 years^e^		0.70 (0.41-1.20)	0.20		
	Proteinuria >3g/d^f^		0.75 (0.38-1.49)	0.42		
	GFR <30ml/min/1.73m^g^		0.59 (0.35-1.00)	0.05		
	Treatment with CYC^h^		2.04 (0.92-4.52)	0.08		

Reference groups: ^a^,GPA; ^b^, ANCA-PR3; ^c^, sclerotic; ^d^, male; ^e^, age <58 years; ^f^, Proteinuria ≤3g/day; ^g^, GFR ≥30ml/min; ^h^ no CYC.

## Data Availability

The data used to support the findings of this study are available from the corresponding author upon request.
